# Supplementary material for: Effects of initial microbial biomass abundance on respiration during pine litter decomposition
Source: PLoS One. 2020 Feb 14;15(2):e0224641. doi: 10.1371/journal.pone.0224641 (PMC7021309; doi:10.1371/journal.pone.0224641)
Supplement: S1 Fig — Estimated biomass for a) bacterial and b) fungi in source community soils using plate counts. (DOCX) [file pone.0224641.s001.docx]

**Figure S1.** Estimated biomass for a) bacterial and b) fungi in source community soils using plate counts.
