# Supplementary material for: Effects of initial microbial biomass abundance on respiration during pine litter decomposition
Source: PLoS One. 2020 Feb 14;15(2):e0224641. doi: 10.1371/journal.pone.0224641 (PMC7021309; doi:10.1371/journal.pone.0224641)
Supplement: S2 Fig — For each boxplot, the line in the box shows the median cumulative CO2, with the endpoints showing the 25% and 75% quartile range. The whiskers show the 0% and 100% quartile range. Separate points outside of whiskers show outliers within a dilution. (DOCX) [file pone.0224641.s002.docx]

**Figure S2.** Cumulative CO_2_ production for all source communtiies after 30 days by relative intial biomass abundance. For each boxplot, the line in the box shows the median cumulative CO_2_ , with the endpoints showing the 25% and 75% quartile range. The whiskers show the 0% and 100% quartile range. Separate points outside of whiskers show outliers within a dilution.
