# Supplementary material for: Effects of initial microbial biomass abundance on respiration during pine litter decomposition
Source: PLoS One. 2020 Feb 14;15(2):e0224641. doi: 10.1371/journal.pone.0224641 (PMC7021309; doi:10.1371/journal.pone.0224641)
Supplement: S3 Fig — a) Cumulative CO2 (+- s.e.) production per day for source community soil 10 and soil 18 in Experiment 2. Initial biomass abundance shown in the same color. Acclimation period is shown with the red bar. Microbial communities were inoculated on 0.2 grams of pine litter, after 2 weeks (priming period), 1.0 grams of litter (5x initial amount) was added. b) Comparison of relative cumulative CO2 (day 30) for each source community based on relative initial biomass (day 0) that was obtained through dilutions. (DOCX) [file pone.0224641.s003.docx]

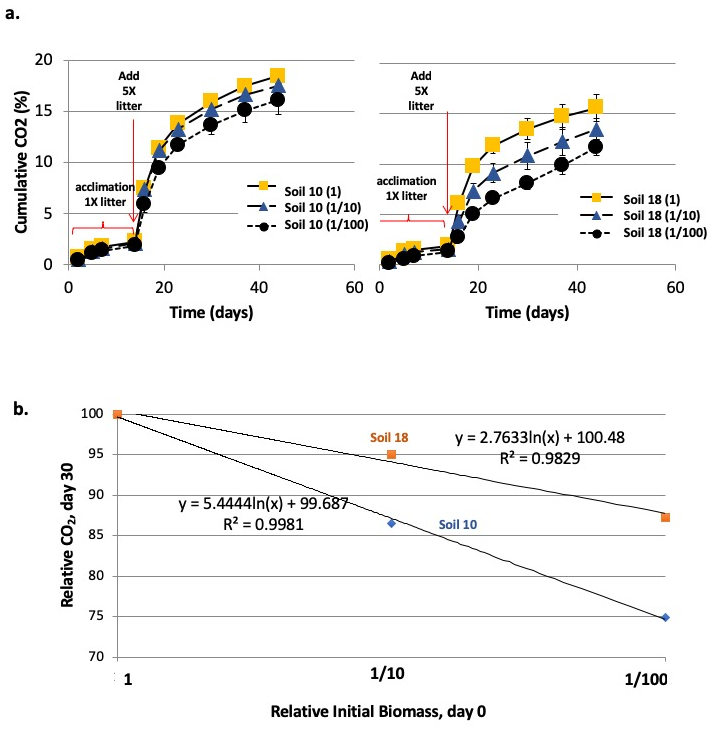


**Figure S3.** **a)** Cumulative CO_2_ (+- s.e.) production per day for source community soil 10 and soil 18 in Experiment 2. Initial biomass abundance shown in the same color. Acclimation period is shown with the red bar. Microbial communities were inoculated on 0.2 grams of pine litter, after 2 weeks (priming period), 1.0 grams of litter (5x initial amount) was added. **b)** Comparison of relative cumulative CO_2_ (day 30) for each source community based on relative initial biomass (day 0) that was obtained through dilutions.
