# Supplementary material for: Effects of initial microbial biomass abundance on respiration during pine litter decomposition
Source: PLoS One. 2020 Feb 14;15(2):e0224641. doi: 10.1371/journal.pone.0224641 (PMC7021309; doi:10.1371/journal.pone.0224641)
Supplement: S4 Fig — Relative abundance of abundant bacterial taxa at the order level, b) Relative abundance of abundant fungal taxa at the genera level. All individual samples are shown for each initial biomass abundance (10−1, 10−2, 10−3). (DOCX) [file pone.0224641.s004.docx]

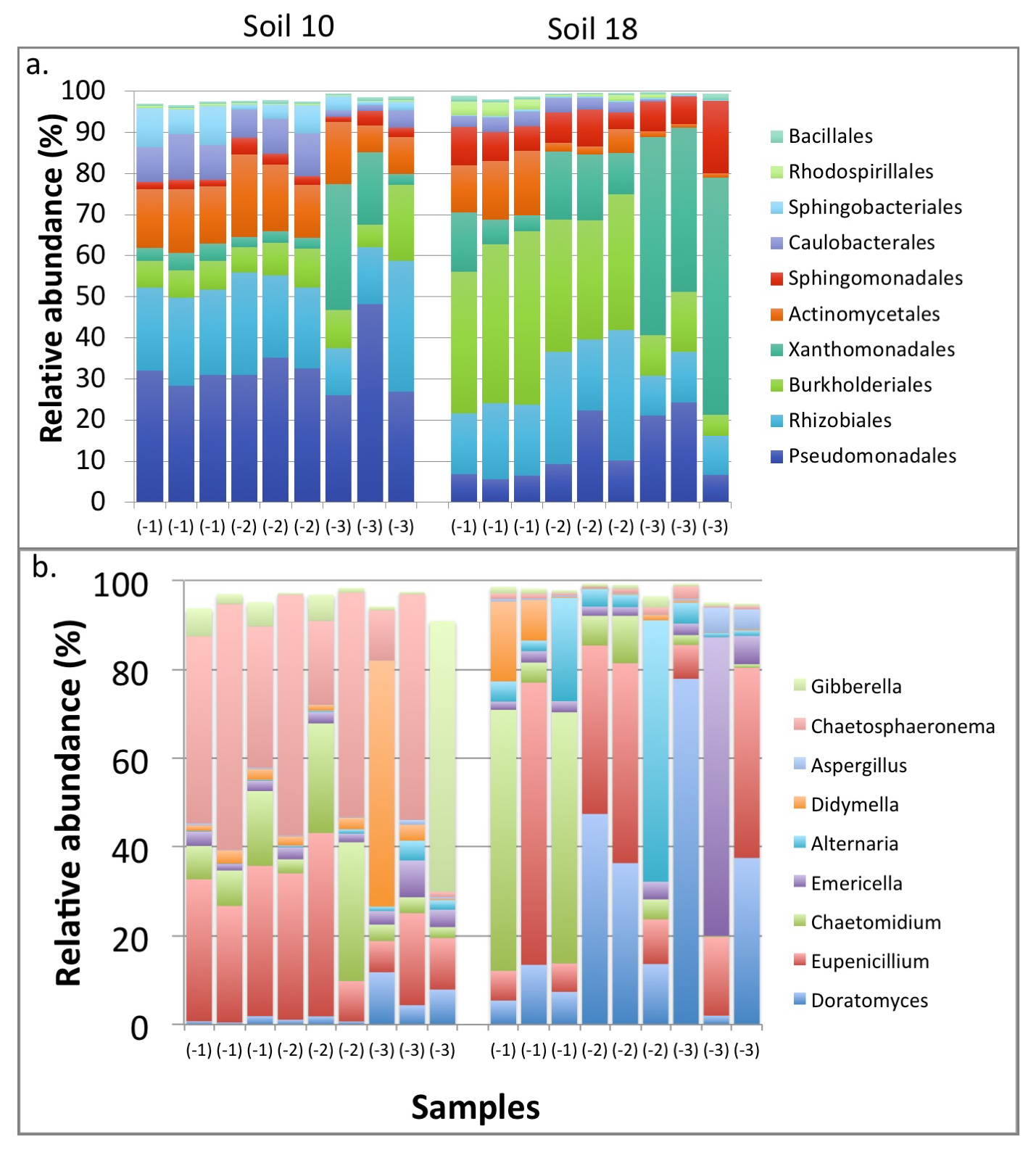


**Figure S4.** Relative abundance of abundant bacterial taxa at the order level, **b)** Relative abundance of abundant fungal taxa at the genera level. All individual samples are shown for each initial biomass abundance (10^-1^, 10^-2^, 10^-3^).
