# Supplementary material for: Effects of initial microbial biomass abundance on respiration during pine litter decomposition
Source: PLoS One. 2020 Feb 14;15(2):e0224641. doi: 10.1371/journal.pone.0224641 (PMC7021309; doi:10.1371/journal.pone.0224641)
Supplement: S1 Table — (DOCX) [file pone.0224641.s005.docx]

**Table S1.** Soils used to extract the twelve source microbial inoculum.

| Soil ID | Longitude | Latitude | Altitude | State |
| --- | --- | --- | --- | --- |
| 010 | -107.72348 | 37.830297 | 9,318 | CO |
| 018 | -111.78211 | 36.709019 | 3,573 | AZ |
| 021 | -105.90896 | 37.993221 | 7,566 | CO |
| 049 | -100.51567 | 35.7576 | 2584 | TX |
| 079 | -103.58482 | 37.9601 | 4219 | CO |
| 096 | -106.0999 | 36.006518 | 5590 | NM |
| 131 | -107.99653 | 36.6571333 | 5629 | NM |
| 135 | -107.1373 | 36.0749667 | 7343 | NM |
| 183 | -109.30587 | 41.49915 | 6589 | WY |
| 201 | -108.77747 | 39.6086667 | 7705 | CO |
| 205 | -108.32643 | 38.8644 | 5191 | CO |
| 215 | -105.89867 | 37.9108167 | 7502 | CO |
