# Supplementary material for: Effects of initial microbial biomass abundance on respiration during pine litter decomposition
Source: PLoS One. 2020 Feb 14;15(2):e0224641. doi: 10.1371/journal.pone.0224641 (PMC7021309; doi:10.1371/journal.pone.0224641)
Supplement: S2 Table — (DOCX) [file pone.0224641.s006.docx]

**Table S2.** Nested ANOVA for richness and diversity metrics and nested Permutational MANOVA for composition metrics.

| **a. Bacterial Richness** |  | |  |  | |  | |  | |  |
| --- | --- | --- | --- | --- | --- | --- | --- | --- | --- | --- |
|  | df | | SS | MS | | F value | | Pr(>F) | |  |
| Source Community | 1 | | 28720 | 28720 | | 25.29 | | **0.0003** | |  |
| Initial Biomass(Source Community) | 4 | | 30879 | 7720 | | 6.798 | | **0.004** | |  |
| Residual | 12 | | 13627 | 1136 | |  | |  | |  |
|  |  | |  |  | |  | |  | |  |
| **b. Bacterial Diversity** | | |  |  | |  | |  | |  |
|  | df | | SS | MS | | F value | | Pr(>F) | |  |
| Source Community | 1 | | 0.954 | 0.954 | | 43.6 | | **2.53E-05** | |  |
| Initial Biomass(Source Community) | 4 | | 4.375 | 1.0939 | | 49.99 | | **2.19E-07** | |  |
| Residual | 12 | | 0.26 | 0.0219 | |  | |  | |  |
|  |  | |  |  | |  | |  | |  |
| **c. Bacteria Community Composition** | | | |  | |  | |  | |  |
|  | df | | SS | MS | | F.Model | | Pr(>F) | |  |
| Source Community | 1 | | 1.5661 | 1.56606 | | 0.47324 | | **0.001** | |  |
| Initial Biomass(Source Community) | 2 | | 0.8165 | 0.40825 | | 0.24674 | | **0.001** | |  |
| Residual | 14 | | 0.9266 | 0.06619 | | 0.28002 | |  | |  |
|  |  | |  |  | |  | |  | |  |
| **d. Fungal Richness** |  | |  |  | |  | |  | |  |
|  | df | | SS | MS | | F value | | Pr(>F) | |  |
| Source Community | 1 | | 84.5 | 84.5 | | 2.461 | | 0.14267 | |  |
| Initial Biomass(Source Community) | 4 | | 1063.1 | 265.78 | | 7.741 | | **0.002** | |  |
| Residual | 12 | | 412 | 34.33 | |  | |  | |  |
|  |  | |  |  | |  | |  | |  |
| **e. Fungal Diversity** |  | |  |  | |  | |  | |  |
|  | df | | SS | MS | | F value | | Pr(>F) | |  |
| Source Community | 1 | | 0.5339 | 0.5339 | | 11.602 | | **0.005** | |  |
| Initial Biomass(Source Community) | 4 | | 0.123 | 0.0307 | | 0.668 | | 0.626 | |  |
| Residual | 12 | | 0.5522 | 0.046 | |  | |  | |  |
| **f. Fungal Community Composition** | |  |  |  | |  | |  | |  |
| df | | | SS | | MS | | F.Model | | Pr(>F) | |
| Source Community | 1 | | 0.8384 | 0.83836 | | 5.0334 | | **0.001** | |  |
| Initial Biomass(Source Community) | 2 | | 0.5645 | 0.28224 | | 1.6945 | | 0.069 | |  |
| Residual | 14 | | 2.3318 | 0.16656 | | 0.62437 | |  | |  |
|  |  | |  |  | |  | |  | |  |
